# Using Phylogeny to Improve Genome-Wide Distant Homology Recognition

**DOI:** 10.1371/journal.pcbi.0030003

**Published:** 2007-01-19

**Authors:** Sanne Abeln, Carlo Teubner, Charlotte M Deane

**Affiliations:** Department of Statistics, University of Oxford, Oxford, United Kingdom; University of California San Diego, United States of America

## Abstract

The gap between the number of known protein sequences and structures continues to widen, particularly as a result of sequencing projects for entire genomes. Recently there have been many attempts to generate structural assignments to all genes on sets of completed genomes using fold-recognition methods. We developed a method that detects false positives made by these genome-wide structural assignment experiments by identifying isolated occurrences. The method was tested using two sets of assignments, generated by SUPERFAMILY and PSI-BLAST, on 150 completed genomes. A phylogeny of these genomes was built and a parsimony algorithm was used to identify isolated occurrences by detecting occurrences that cause a gain at leaf level. Isolated occurrences tend to have high e-values, and in both sets of assignments, a sudden increase in isolated occurrences is observed for e-values >10^−8^ for SUPERFAMILY and >10^−4^ for PSI-BLAST. Conditions to predict false positives are based on these results. Independent tests confirm that the predicted false positives are indeed more likely to be incorrectly assigned. Evaluation of the predicted false positives also showed that the accuracy of profile-based fold-recognition methods might depend on secondary structure content and sequence length. We show that false positives generated by fold-recognition methods can be identified by considering structural occurrence patterns on completed genomes; occurrences that are isolated within the phylogeny tend to be less reliable. The method provides a new independent way to examine the quality of fold assignments and may be used to improve the output of any genome-wide fold assignment method.

## Author Summary

When predicting the structure for a protein sequence, a major step is achieved when an evolutionarily related protein with a known structure is identified. This process is called fold recognition, and has been a major force behind improvement in structure prediction. Moreover, fold-recognition techniques have become increasingly important in recent years because of the huge numbers of protein sequences with unknown structures available through sequencing projects on completed genomes. However, all fold-recognition methods tend to produce either a large number of false negatives (at high confidence scores) or a large number of false positives (at low confidence scores). Here we show that the reliability of a fold-recognition technique can be explored by analysing its predictions across a set of completed genomes. We have developed a method that can indicate false positives in these genome-wide assignment sets. The basic idea behind the method is that a fold assignment on a genome is less reliable if the prediction is not observed on evolutionary related genomes. The ability of the method to discriminate false positives is confirmed by independent tests. The method can be used on the output of any genome-wide fold assignment method.

## Introduction

The prediction of protein structures from sequences is becoming increasingly important, particularly as the gap between the number of experimentally determined sequences (>6,000,000) and structures (<35,000) widens. Knowledge of protein structure is essential to the understanding of biochemical processes. In practical terms, knowledge and prediction of protein structures can aid the discovery of new drugs [1].

When predicting the structure for a target sequence, a major step is achieved when an evolutionarily related protein with a known structure is identified. Since structure is more conserved than sequence, it is presumed that the target sequence has a similar fold to the related protein. This process is called fold recognition and has been a major force behind improvement in structure prediction in recent years [2].

At present there are several ways to recognise a fold for a given sequence. If there is close homology between the target sequence and a known structure in the Protein Data Bank (PDB) [3], a simple sequence search, such as BLAST [4], will be sufficient to identify the fold. To detect more distant homologies we can use position-specific scoring methods such as PSI-BLAST [5] and hidden Markov model (HMM)–based methods such as SAM-T98 [6]. These methods are the least expensive forms of fold recognition and are sequence based. There are more computationally expensive methods that take structural information into account; an example of such a technique is THREADER [7].

Recently, many studies have used fold recognition to look at the structural content of entire genomes with aid of PSI-BLAST [8], HMMs [9,10], or threading procedures [11]. These fold-recognition assignments can produce an occurrence pattern on a set of species for a given family, superfamily, or fold as defined by structural classifications such as SCOP [12] or CATH [13]. Sets of such occurrence patterns have proved to be useful for building a phylogeny of species [14,15], for grouping proteins within a similar pathway [16], and for estimating the ages of folds [17].

A major challenge for all fold-recognition techniques is to discriminate a true homologue from a false positive (specificity) using confidence scores such as e-values. e-Values (expectation values) indicate how likely it is that an alignment with the search sequence would occur by chance in a given database (i.e., they should reflect the chance of a false positive assignment). Previous studies have suggested that analysis of structural assignments on completed genomes may indicate false positives of fold-recognition techniques. Yang and coworkers [15] showed that the number of hits on completed genomes drastically increased above a certain e-value cutoff, which could be explained by a sudden influx of false positives. Furthermore, Winstanley and coworkers [17] showed that occurrence patterns of most superfamilies can explain the phylogeny of the genomes reasonably well, but observed a difference in fitness between patterns from two different fold-recognition techniques. In particular it appeared that, in one set, more assignments were made that occurred on isolated leaves of the species tree.

We propose that false positives in fold-recognition assignments might be identified by considering a phylogeny of species. False assignments in such a set might be expected to occur randomly across the genome tree, whereas true positive assignments to a superfamily should be evolutionary related. Hence, we expect that false occurrences have a stronger tendency to be scattered across the tree than true assignments. This study investigates whether isolated occurrences within a phylogenetic occurrence pattern are indeed more likely to be false positives.

Using phylogeny to improve homology searches is not a new idea. It has been known for a long time that by considering the phylogeny of related proteins one can improve sequence alignments. An example is progressive multiple sequence alignment, where an approximate phylogeny of the sequences is used to aid the alignment of multiple sequences [18].

Assignment of function can be facilitated by phylogenomics: a set of known homologs is used to create a phylogeny of proteins in which speciation and duplication events are marked. These can be used to subclassify the proteins in the phylogeny into specific functions. Several protocols as well as automated procedures based on phylogenomics have been able to improve functional annotation [19–21]. Recently it has also become clear that confidence in a modelled structure increases when homologues of the target sequence give a similar structure prediction [22,23]. Here, precalculated phylogenies of entire genomes are used rather than phylogenies of individual proteins.

In this study we will assess genome-wide assignments obtained by PSI-BLAST searches as well as assignments from the SUPERFAMILY database. PSI-BLAST is an iterative version of BLAST, which uses a position-specific scoring matrix (PSSM) to include information about homologous sequences. The PSSM is used to identify the amino acids that are most likely to occur at a given position in the sequence. During each run, sequence information from all hits, with an e-value below a threshold, is added to the PSSM. Final e-values between the target and each database sequence are based on sequence similarity to the PSSM, which is subsequently normalised for amino acid composition with respect to the entire database.

The SUPERFAMILY database [9] is built with SAM-T99 [6], a procedure to find distant homologues using HMMs. The SAM-T99 program was finetuned with expert knowledge to recognise superfamilies as defined by SCOP. For each search sequence a profile HMM is created from homologues, which are found by a simpler sequence similarity search. The HMM is a statistical model that describes the evolutionary behaviour for a set of homologous sequences. These models are then used to calculate a score for each sequence in the database. The e-values are normalised by the reversed score of the searching sequence.

To assess the assignments, occurrence patterns were obtained for every superfamily from our two fold-recognition sets (PSI-BLAST and SUPERFAMILY). We developed a method that identifies isolated occurrences within such occurrence patterns by considering if an occurrence causes a gain at leaf level in the phylogeny.

This study demonstrates that false positives in fold-recognition assignments can indeed be identified by considering a phylogeny of species: isolated occurrences are shown to have higher e-values (are less reliable) than other occurrences. We formulated criteria to predict false positives based upon these results. The set of predicted false positives were validated by comparisons to overlapping PSI-BLAST assignments and to assignments that changed between different versions of the SUPERFAMILY database. Both tests confirmed that the predicted false positives are far more likely to be falsely assigned than other occurrences.

Analysis of occurrence patterns from genome-wide fold recognition also provides a new way to examine the quality of fold assignments. We show that the frequency of occurrences drastically increases for high e-values (>10^−8^ for SUPERFAMILY and >10^−4^ for PSI-BLAST), and that this influx is likely to be caused by false positive assignments. In addition, the accuracy of the fold recognition is demonstrated to differ significantly for the different structural classes as defined by SCOP. In principle, this technique can screen assignments of any existing fold-recognition method for false positives. An extended version of the method is given, which can be applied to assignment sets with a high proportion of false positives. This version might be able to improve the search capacity of any genome-wide fold-recognition technique.

## Results

### Phylogeny and Accuracy of Assignments

#### Assignments.

All assignments were generated by searching for SCOP domains on the genes of 150 completed genomes (18 archaea, 97 bacteria, and 35 eukaryotes; see Table S1).

The first set of assignments was taken from the SUPERFAMILY database [24] and contained 1,269 different superfamilies. The database provided about 750,000 structural assignments for our set of genomes (Table 1). One or more structural assignments was made to 48% of the gene sequences in this dataset, with a false positive rate estimated to be lower than 1% [24].

**Table 1:**
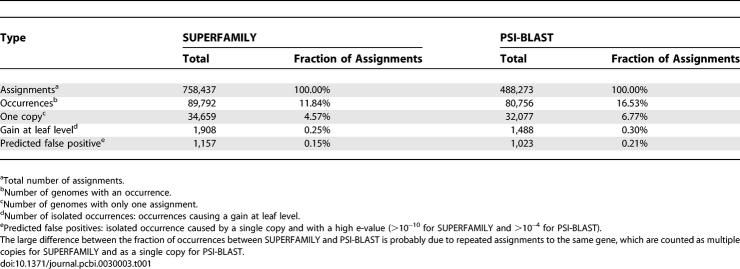
Fraction of Assignments That Can Be Analysed by the Method

The second set consisted of assignments obtained using PSI-BLAST [5]. It included all assignments with an e-value lower than 1.0 for testing purposes. The inclusion threshold to build the PSSM was kept low, at 10^−5^, to diminish the risk of including false positives within the search profile. About 450,000 structural assignments were made with PSI-BLAST (Table 1). As expected, the coverage of genes was a little lower, with one or more structural assignments made to about 36% of the genes. For more details on both sets of assignments, see Materials and Methods.

#### Parsimony and gains at leaf level.

**Figure 1:**
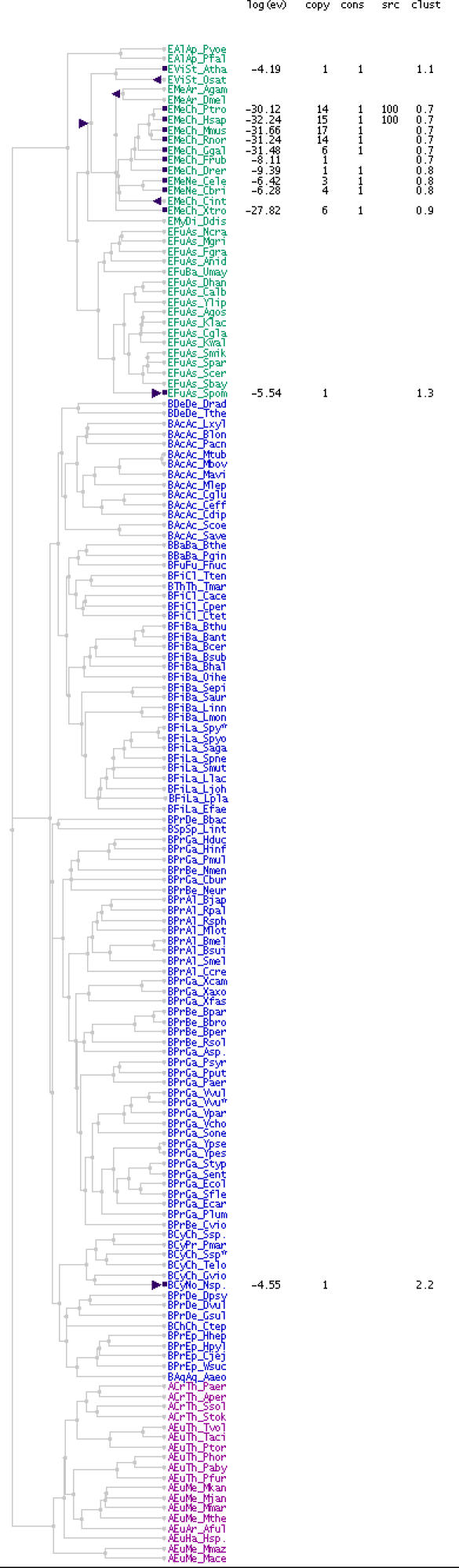
The Phylogeny, Including All 150 Genomes, Showing the Occurrence Pattern for Superfamily a.126.1 (Serum Albumin-Like) for SUPERFAMILY Data Losses (left-pointing triangles) and gains (right-pointing triangles) are shown as calculated by the parsimony algorithm. The assignments with a gain at leaf level are isolated within the tree. For each occurrence the log10 e-value (log(ev)), the number of copies (copy), consensus with PSI-BLAST occurrence (cons), and cluster distance (clust) are shown. The src column shows which genomes contain a source sequence for the superfamily by indicating the sequence identity to a SCOP domain.

We first calculated a phylogeny for the genomes. Occurrence patterns were used to build the tree; this has previously been shown to be more robust than tree-building using the number of assignments or copies [14,15,17]. Figure 1 shows the phylogeny used (see Materials and Methods for more details on the tree-building process).

A parsimony algorithm was used to predict an evolutionary scenario of loss and gain events for a superfamily given this phylogeny. The algorithm minimises the number of loss and gain events in the tree for the occurrence pattern of the superfamily.

Our measure for isolatedness indicates whether an occurrence causes a gain at leaf level in the set of minimal gains and losses. The parsimony algorithm predicts a gain at leaf level (gain at the lowest level of the tree), when it would be more expensive to cluster the occurrence with other occurrences. Hence, an assignment with a gain at leaf level is an isolated occurrence within the pattern. Note that the parsimony algorithm makes the sets of loss and gain events quite robust against small changes in the occurrence pattern. A few deletions within an occurrence cluster will usually not cause a gain at leaf level; this diminishes the effect of deletions and false negatives.

Figure 1 shows the full assignment of gains and losses by our parsimony algorithm for superfamily a.126.1 (serum albumin-like).

#### Detectable false positives.

The technique developed here can only observe false positive occurrences. However, the same superfamily can be assigned to many different genes on a genome and so one occurrence might cover many assignments. As the number of assignments (or copies) for a superfamily on a genome is known to behave like a power law [8,14], the number of genomic occurrences is much smaller than the number of assignments. In this technique, the e-value for an occurrence is determined by the lowest e-value in the set of copies on the genome covering the occurrence. In effect, if a superfamily has many copies on a genome, one of the assignments usually has a very low e-value, which will overshadow those with higher e-values. This effect obviously reduces the number of assignments that can be screened by our technique (Table 1).

In practise we will restrict the set of predicted false positives to isolated occurrences caused by one copy with a high e-value (see below).

#### E-value distributions.

To understand the e-value distribution of isolated occurrences, we first consider the e-value distribution of all assignments and the e-value distribution of all occurrences.

**Figure 2:**
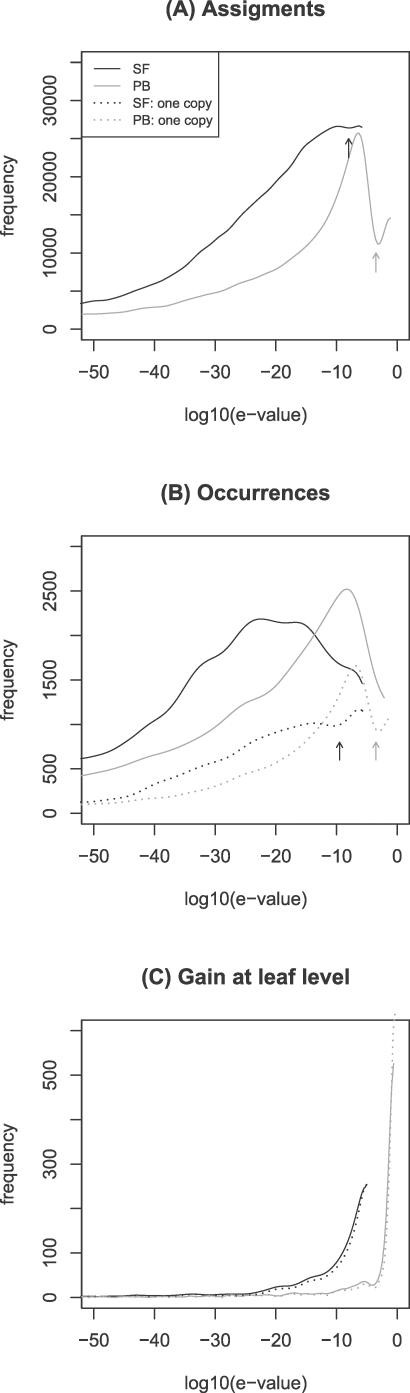
e-Value Distributions for SUPERFAMILY (SF) and PSI-BLAST (PB) Assignments and Occurrences (A) e-Value distribution of all assignments. (B) e-Value distribution of occurrences. The e-value of an occurrence is defined as the lowest e-value of all assignments to a superfamily on a genome. (C) e-Value distribution of occurrences that cause a gain at leaf level (i.e., predicted false positives). Arrows indicate local minima in the distribution, which might indicate the point where false positive assignments become more dominant.

Figure 2A shows the distribution of e-values for all fold assignments. A local minimum is seen at the higher e-value end of the distribution for both the SUPERFAMILY and PSI-BLAST distributions. This may mark the point where false positives assignments begin to play an important role. Figure 2B shows the e-value distributions for all occurrences. This distribution is shifted to the left, with respect to the assignment distribution.

The e-value for an occurrence is taken from the assignment on a genome with the lowest e-value; hence, assignments with higher e-values from the same genome are not represented in this set. The left shift is, as expected, not seen for occurrences with a single assignment on a genome (dotted lines in Figure 2B), since the effect described above cannot occur for occurrences caused by only one assignment. In fact, the one-copy distribution appears to be very similar to the overall assignment distribution. The fraction of false positive assignments should be very small; false positive occurrences would therefore generally be caused by a single false assignment on a genome. Hence, the fraction of false positives in the one-copy distributions is expected to be higher than the fraction of false positives for all assignments. Indeed, it is observed that the local minima have become more prominent in these distributions.

#### False positives.

We now compare two independent sets of observations: (1) a set of binary values indicating if an occurrence causes a gain at leaf level; and (2) a set of continuous e-values for every occurrence (for discussion on independence see Evolutionary Caveats). e-Values should indicate how likely it is that an occurrence is a false positive. We can therefore use the e-values to investigate our expectation that isolated occurrences are more likely to be generated by a false assignment.

Figure 2C shows the e-value distribution for occurrences with a gain at leaf level. Comparing it with the other distributions, it is clear that this distribution is shifted to the right. This shift appears to confirm the supposition that occurrences with a gain at leaf level are less reliable. The steep increase in the distribution for the SUPERFAMILY data starts at a significantly lower e-value than for PSI-BLAST, possibly indicating that false positives in the SUPERFAMILY set start occurring at lower e-values. However, the e-values for the two sets of assignments are not necessarily comparable, since, as we described earlier, they are calculated using different estimation techniques.

#### Ranked distributions.

The e-value distributions for the assignments on the genomes are clearly not normally distributed. To make a comparison between different e-value distributions simpler, we have given a rank to the e-value of each assignment. This ranked e-value distribution of all assignments gives a uniform distribution, which would display as a flat horizontal line (not shown). Similarly, any random subset of this reference distribution is expected to be uniform.

**Figure 3:**
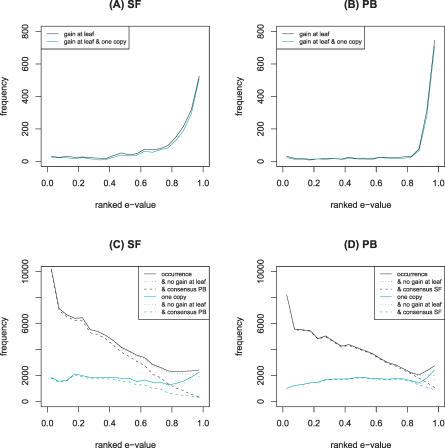
Ranked e-Value Distributions for SUPERFAMILY (SF) and PSI-BLAST (PB) Occurrences The ranked e-value is obtained by ranking the e-values in the set of all assignments (including multiple copies) and is normalised between 0 and 1. (A,B) Ranked e-value distributions for occurrences with a gain at leaf level. The peak at the righthand side might indicate a sudden increase in false positives. (C,D) Ranked e-value distribution for all occurrences. The dotted line indicates that occurrences with a gain at leaf level have been removed, and the striped line shows only consensus occurrences, which have been assigned by both SUPERFAMILY and PSI-BLAST. Occurrences caused by a single assignment (one copy) are shown in colour. Both the distributions for all occurrences and for occurrences caused by a single assignment show an increase at the righthand side, probably caused by false positives. This increase is not seen in distributions either where either predicted false positives are taken away or for consensus occurrences.

Figure 3A and 3B show that the left-hand side of the distribution of occurrences with a gain at leaf level is approximately uniform and hence can be interpreted as a random subset of the assignment distribution. However, for higher e-values, a sudden increase is observed; this indicates that relatively more assignments with a high e-value are found in occurrences with a gain at leaf level than in the reference distribution. We believe that this set of additional isolated occurrences at high e-values is caused by false positive assignments.

The ranked e-value distribution for the set of all occurrences (black line) in Figure 3C and 3D is, as expected, shifted to the left with respect to the reference distribution; the e-value of an occurrence is defined as the best e-value on the genome.

However, at higher e-values, the distribution stops decreasing, and even shows an increase for PSI-BLAST. Moreover, the distribution of consensus occurrences, which are predicted by both SUPERFAMILY and PSI-BLAST assignments, does not show a sudden increase at high e-values (striped line). As it has been reported that the consensus of two or more fold-recognition methods can significantly improve the specificity of individual methods [25], the observed increase is likely to be an artefact because of a sudden appearance of false positives for high e-values.

If the set of “gains at leaf level” is taken away from this ranked occurrence distribution (dotted line in Figure 3), the distribution continues to decrease as expected. This shows that the set of isolated occurrences corresponds in size and e-value distribution to a set of likely false positive occurrences.

As described above, occurrences caused by only one assignment should have a similar distribution to that of all assignments, and, hence, the ranked distribution should appear uniform. The coloured line in Figure 3C shows that this is roughly the case for both the SUPERFAMILY and PSI-BLAST occurrences. However, both distributions show a sudden increase at high e-values. Once again, the distribution of consensus occurrences does not show such an increase (coloured striped line). The set of single occurrences from which isolated occurrences have been removed (coloured dotted line) shows less of an increase than the occurrence distribution.

These results all support that the observed change in distribution for high e-values is created by a sudden increase in false positives, and that our set of isolated occurrences corresponds to these false positives. The results also demonstrate that occurrences with a gain at leaf level have in general worse e-values than other assignments.

### Evolutionary Caveats

We used fold-recognition methods to find members of a superfamily. Hence, all assignments and occurrences within a pattern should be evolutionarily related. We propose that an isolated occurrence on a species tree might indicate that that occurrence is a false positive. Below we consider what other mechanisms might cause a bad fit within an occurrence pattern for a superfamily.

#### Horizontal gene transfer.

An isolated occurrence within the tree might be due to horizontal gene transfer (HGT) between species. This is known to be an important process for the enrichment of diversity on genomes [26]. However, we currently have no reason to believe that instances of HGT would give higher e-values for true superfamily assignments than their non-HGT counterparts. We will in practise only consider isolated occurrences as a false positive when the e-value of the occurrence lies above a certain threshold. These thresholds should match values at which we observe a sudden increase in frequency for the ranked e-values (10^−10^ for SUPERFAMILY and 10^−4^ for PSI-BLAST). Vice versa, the extent of HGT might be estimated by the part of the distribution for occurrences with a gain at leaf level, which is independent of e-values. In the ranked e-value distributions, this is the uniform part (Figure 3A and 3B).

#### Distance to source sequence.

Another possible source of error in the technique is a dependency of e-values on the evolutionary distance between the search sequence and an assignment. The search sequence is the initial sequence from which a search profile (HMM/PSSM) is created. This dependence is likely to be smaller for profile-based methods than for simple sequence search tools such as BLAST. In this study, the search sequences are the “source” domains taken from SCOP.

We have so far assumed independence of e-values and phylogenetic fitness of an occurrence. The above effect would not cause a direct dependence, but together with deletion of domains, false negatives, and random noise in e-values, it could result in higher e-values close to the edges of an occurrence cluster. Two different tests were performed to see if our independence assumption would hold.

First, we examined the relationship between e-values and the distance from an occurrence to its source sequence. A genome is said to contain a source sequence for a superfamily when a very close match to a sequence of a known 3-D structure is found in its genes (see Materials and Methods). The distance to a source was calculated as the height of the lowest common ancestor in the tree between the genome of the occurrence and any of the genomes containing a source sequence for the superfamily.

There exists a very weak correlation between the distance to a source sequence and the e-value of an occurrence, with r^2^ values of 0.05 for SUPERFAMILY and 0.14 for PSI-BLAST (see Figure S1). The correlation becomes even weaker (r^2^ = 0.04 and 0.10) when the set of source occurrences is removed. Despite the correlations being very weak, they are significant (*p* < 2e^−16^) for both PSI-BLAST and SUPERFAMILY.

To assess the influence of this very weak correlation on our method, we examined whether e-values deteriorated towards the edges of a cluster. The cluster distance of an occurrence is calculated as the distance to every leaf in the occurrence pattern divided by the distance to every leaf in the tree. It is then mediated so that the cluster distance of an occurrence is relative to its pattern. Scores significantly higher than 1.0 reflect bad clustering of an occurrence in the tree.

**Figure 4:**
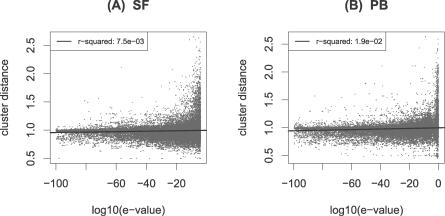
e-Value versus Cluster Distance for SUPERFAMILY (SF) and PSI-BLAST (PB) (A,B) Show there is no correlation between the e-value and cluster distance. The cluster distance indicates whether an occurrence lies within a cluster of occurrences (see Materials and Methods for more details).

Figure 4A and 4B show that there is a negligible correlation between the cluster distance and the e-value of an occurrence, and in general we can assume independence of these entities (see Figure S2). This allows us to rule out the possibility that a general correlation between cluster distance and e-values causes the previously observed increase in isolated occurrence at very high e-values.

### Saturation Effects on Patterns

The more occurrences there are in a pattern, the lower the chance that an isolated occurrence can appear. Therefore, if a pattern saturates with occurrences, fewer false positives can be predicted through our gain at leaf level technique. There is a strong anticorrelation (r^2^ = 0.95) between the number of occurrences in a pattern and potential gains at leaf level. A potential gain at leaf level is a leaf without an occurrence, which would cause a gain at leaf level when added to the pattern.

Occurrence patterns can become saturated through an increase in either true or false positive assignments (see Discussion for consequences of additional true assignments). An increase of false positive occurrences could be created by the inclusion of very high e-values into the set of assignments. This might be desirable to enlarge the capacity of existing fold-recognition methods. However, if false occurrences start to dominate the pattern, our technique would begin to fail.

The problem can be overcome using a slight modification to the technique. The parsimony algorithm is first run on a base pattern that is created from assignments below a strict e-value threshold. Genomes without an occurrence in this base pattern are checked for potential gains at leaf level. Then the set of isolated occurrences becomes the union of all gains at leaf level in the base pattern and all potential gains at leaf level, which have an occurrence with an e-value above the threshold. This modification could cause the algorithm to overpredict the number of false positives if the e-value cutoff is set too low.

**Figure 5:**
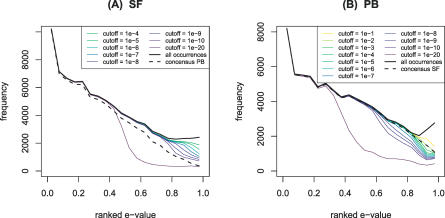
Ranked e-Value Distributions for Occurrences without Isolated Occurrences The predicted false positives are calculated through a base pattern created from assignments with an e-value below the threshold. If an occurrence lies on a genome with either a gain at leaf level in the base pattern or a potential gain at leaf level, it is here defined as an isolated occurrence. A clear overprediction of isolated occurrences is only seen in the set with a base pattern cutoff of 10^−20^.

Figure 5 shows the results of running this procedure with different e-value cutoffs for the base pattern. Only when using extremely low e-value cutoffs (e.g., 10^−20^) are a considerable number of isolated occurrences detected for middle-range e-values. Hence, the above method can potentially be used without a huge overprediction of false positives. In fact, comparing the distributions (coloured lines) to the consensus data (striped line), a sensible threshold can be determined with an e-value cutoff of 10^−8^ for SUPERFAMILY and 10^−2^ for PSI-BLAST. This version of our method can be used on occurrence pattern sets with a high proportion of false positives.

### Fold Recognition and Structural Classes

At the highest level of the SCOP hierarchy, domains are grouped into seven different classes, predominantly based on secondary structure content. In our analysis, predicted false positives rates for these structural classes differ significantly from one another. For example, the first column in Table 2 shows that the proportion of occurrences with a gain at leaf level is lower for the alpha/beta and multidomain classes than for the all-alpha, all-beta, transmembrane, and small protein classes (SUPERFAMILY).

**Table 2:**
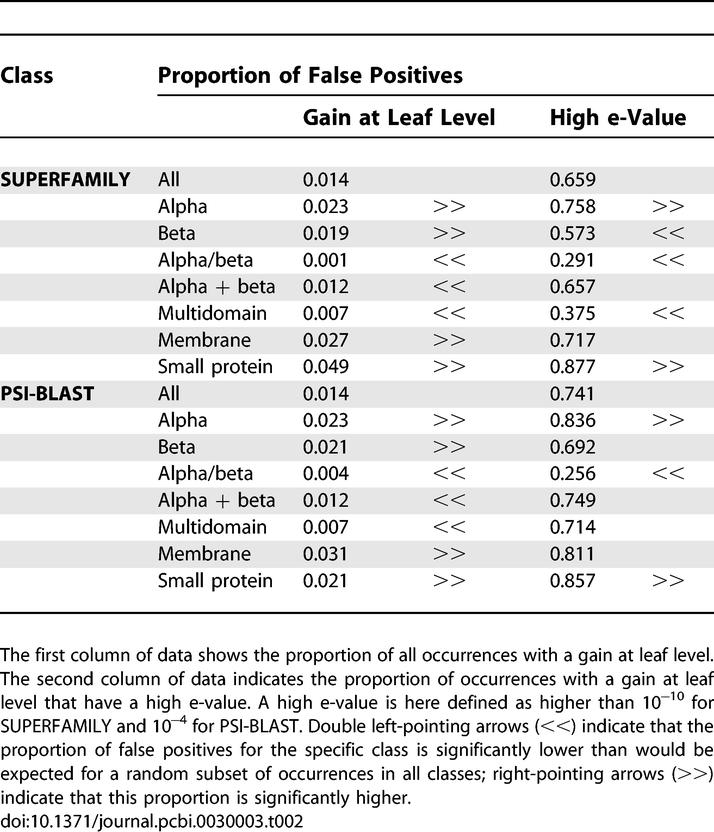
Proportion of Predicted False Positive Assignments for Structural Classes

Some of these differences, although not all, might be explained by the average chain length of the proteins. Domains from the alpha/beta and multidomain classes have on average longer chain lengths than domains from the all-alpha and small-protein classes. Karplus and coworkers [6] previously observed that HMM-based methods might be less accurate at estimating correct e-values for small sequences; this could result in a higher false positive rate. Note that the multidomain class is different from the other six, as it contains proteins that cannot yet be split up into separate domains based on SCOP classification rules. This class is included here because it shows some evidence for sequence-length dependence.

To investigate in more detail why certain structural classes have higher rates of predicted false positives, we submitted a small number of predicted false-positive gene regions to the meta servers (http://bioinfo.pl/meta and http://genesilico.pl/meta). The servers predicted quite a few of the predicted alpha-class regions to be in coil-like structures. On inspection of the source domain, we observed that the region which generated the assignment often contained a very long alpha helix. This strong helical–helical scoring may explain the slightly higher false positive rate for the all-alpha class.

These results must be interpreted with a little care, as the proportions of isolated occurrences might correlate with saturation of the occurrence patterns. As described above, more saturated occurrence patterns result in fewer potential gains at leaf level. Domains from the class of small proteins have on average a lower age than domains from the alpha/beta class [8,17], and will therefore have on average emptier occurrence patterns. To correct for this, the second column of Table 2 shows the proportion of occurrences with a gain at leaf level that have a high e-value (>10^−10^ for SUPERFAMILY and >10^−4^ for PSI-BLAST). These proportions indicate that the all-alpha and small-protein classes still display a higher proportion of false positives than the alpha/beta class for both the SUPERFAMILY and PSI-BLAST assignments.

The second score seems to deviate more per class for SUPERFAMILY than for PSI-BLAST, while the standard deviations for the sample distributions are very similar. This may indicate that there is a higher e-value dependence on secondary structure content or domain length for the former method.

### Validation

Conventional benchmarking of fold-recognition methods involves blind tests on subsets of protein domains with a known structure [27–29] or on sets of sequences annotated with expert knowledge [30]. Using such a procedure, the ratio of false predictions can be estimated as well as the ratio of false negatives. A similar fashion of benchmarking would be difficult for our method, since the majority of genes have unknown structures, and using all genes on the genomes is essential to the method. We have already shown through e-value distributions that isolated occurrences are less reliable. Below we describe two other independent tests to verify our set of predicted false positives. Predicted false positives are defined as an occurrence with a gain at leaf level, a single copy, and a high e-value (>10^−10^ for SUPERFAMILY and >10^−4^ for PSI-BLAST).

Although no actual structures are available for the majority of the genes, a set of likely false positive assignments can be obtained by checking for overlap. For a given gene region, more than one assignment may be obtained. If the assignments are to two different superfamilies, the assignment with the worse e-value is likely to be false.

Translating this to occurrence patterns, we can say that occurrences, which are solely generated by assignments with a stronger assignment in the same region, are likely to be false. We selected these occurrences and compared them with our sets of predicted false positives. Table 3 shows that the overlapping occurrences are found nine times more often in our set of predicted false positives than in general occurrences. Fisher's exact test confirms that this difference in numbers is highly significant (*p* < 2.2 × 10^−16^). Moreover, if the ratio of overlapping regions is measured within a set of occurrences above the same e-value threshold as the set of predicted false positives and is compared with this ratio of the set of predicted false positives, the fraction of overlapping regions remains significantly higher for the predicted false positives (*p* = 2.7 × 10^−5^).

**Table 3:**
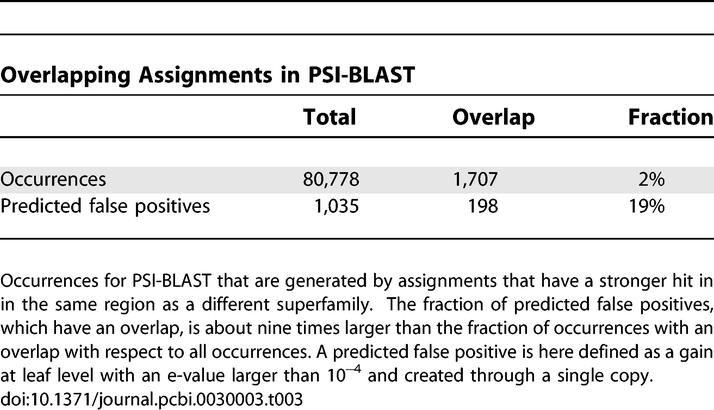
Overlapping Regions in PSI-BLAST

Unfortunately, this test can only be carried out on PSI-BLAST assignments, because in the SUPERFAMILY database, overlapping regions have already been removed. Instead we can compare two different versions of the database. Our study has been carried out on SUPERFAMILY version 1.65. The more recent version (1.69) is deemed to be more reliable.

We assessed assignments that were present in SUPERFAMILY version 1.65, but which had been removed in version 1.69. Again, we selected occurrences for which all assignments on the genome were removed (Table 4). More than 90% of our predicted false positives in SUPERFAMILY 1.65 had been removed in version 1.69. The fraction of removed occurrences is significantly higher in the set of false positives than in general occurrence (*p* < 2.2 × 10^−16^) and significantly higher than in the set of occurrences with a similar e-value (*p* < 2.2 × 10^−16^).

**Table 4:**
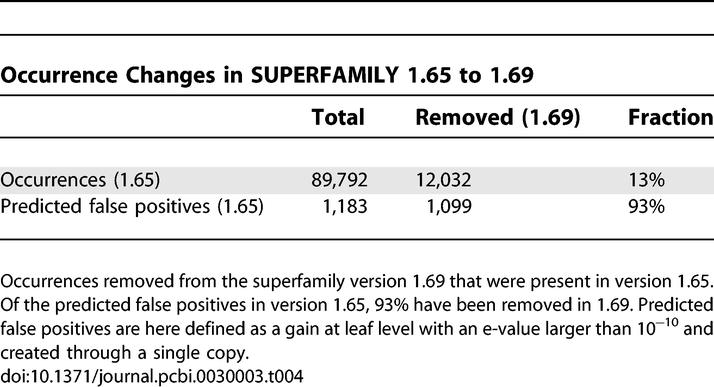
Updates in the Superfamily Database

These results both confirm that our set of predicted false positives are significantly more likely to be false than general occurrences.

## Discussion

### An Increase in True Positives

As described above, fewer isolated occurrences can be detected as occurrence patterns become more saturated. Such saturation could be caused by an increase in true positive assignments, when fold-recognition techniques become far more sensitive, or when many more protein structures become available. Previous work [8] has shown that the number of occurrences is not uniformly distributed. The distribution peaks at a low and a high number of occurrences, with a minimum in the middle. In addition, recent work by Yan and Moult [31] shows that the number of known (super)families, which occur on very few genomes, is expected to grow. This implies that although some superfamily patterns might become more saturated, the number of superfamilies with emptier patterns will also grow. Hence, this method will remain applicable to a large number of superfamilies.

### Biological Relevance

When interested in the fold assignment of a single protein, it is important to keep in mind that an isolated occurrence may appear due to lateral gene transfer or extensive gene loss. Nevertheless, it can be advantageous to visually inspect predicted occurrence patterns of homologous sequences with a weak hit to the protein of interest. Anomalies in such a pattern can give an idea about the reliability of the assignments, and may also indicate false negatives or deletions in cases of a “loss at leaf level.”

When working on a specific protein, it is also important to be aware of the differences in aim and methodology between this method and phylogenomics methods used for functional annotation. The aim of this study is to find true homologues in a set of likely homologues, whereas phylogenomic methods aim to subclassify a set of known homologues. The difference in aim results in a distinctively different methodology. For known (close) homologues, it is feasible to calculate a phylogeny of proteins and subsequently assign duplication and speciation events [21], whereas in this work, the proteins of interest have very distant (if any) evolutionary relationships and a precalculated phylogeny of genomes is used instead. Hence, our method is not a substitute for a phylogenomics method, but could perhaps be used as a prefilter in cases where structural assignments are involved (e.g., see Sjolander [20]).

### Consensus Data and Meta Servers

Previously we described how the method could be extended to assess assignment sets with a large proportion of false positives using a base pattern. However, rather than assignments below an e-value threshold, consensus occurrences could be used to create the base pattern. In this study, two profile-based searching methods were analysed. Although a large proportion of occurrences is in agreement (84% for SUPERFAMILY and 95% for PSI-BLAST), only about 50% of these occurrences were caused by the same number of assignments. The two fold-recognition methods might recognise different homologues on the same genome. Consensus occurrences appear to be more reliable on evaluation by our method: the proportion of isolated occurrences was significantly lower than for occurrences obtained by a single method, even though consensus occurrence patterns are naturally sparser. The usage of consensus occurrences rather than consensus assignments could therefore provide additional information about the reliability of the assignments. This technique might be used as an additional quality check for meta servers, using consensus data of several genome-wide fold-recognition methods.

### Conclusions

This study shows that false positives assigned by fold-recognition methods on completed genomes can be detected by determining isolated occurrences in a phylogeny. We developed a method to identify these isolated occurrences by applying a parsimony algorithm to a phylogenetic occurrence pattern. An occurrence is said to be isolated if it causes a “gain at leaf level” in the most parsimonious evolutionary scenario.

It is shown that in principle, isolated occurrences are less reliable than other assignments: the majority of isolated occurrences have a high e-value (>10^−8^ for SUPERFAMILY, >10^−4^ for PSI-BLAST). e-Values are shown to be almost independent of evolutionary distance between the source sequence and the genome of assignment. Deletions and/or false negatives are therefore unlikely to cause the observed high e-values of isolated occurrences. To predict false positives in practise, additional constraints should be imposed. The e-value of the occurrence should be higher than a given e-value (>10^−8^ for SUPERFAMILY, >10^−4^ for PSI-BLAST) to minimise the number of isolated occurrences caused by lateral gene transfer. In addition, the occurrence (of a superfamily) should be caused by a single assignment to the genome. Using this technique, more than 1,000 false positives can be predicted for both SUPERFAMILY and PSI-BLAST. Tests with independent means to indicate false positives were performed to validate our predicted false positives; one test was based on overlap of PSI-BLAST assignments, and the other considered changes between different versions of the SUPERFAMILY database. Both tests confirmed that isolated occurrences are more likely to be falsely assigned. The method can be extended to assess sets of assignments with a large proportion of false positives and could be used to enhance the searching power of existing fold-recognition techniques. This technique could therefore provide a way to fundamentally improve the assignment sets of genome-wide fold recognition.

Considering occurrence patterns from genome-wide fold recognition also gives a new way to examine the quality of fold assignments. It was observed that the number of assignments and occurrences on genomes drastically increases for high e-values. The most likely explanation for this phenomenon is a sudden increase in false positive assignments above certain e-values, since the sudden increase is not observed in consensus data, with occurrences predicted by both SUPERFAMILY and PSI-BLAST assignments, or for data where isolated occurrences were removed.

When examining the rate of predicted false positives for different structural classes, a significant variance was observed. Domain length and secondary structure content might cause this dependency between false positive rate and structural class.

## Materials and Methods

### SUPERFAMILY assignments.

The first set of assignments was taken from SUPERFAMILY database [24] version 1.65 and covers 1,269 different superfamilies as defined by SCOP [12] on 150 completely sequenced genomes. About 750,000 structural assignments were made, with the following restrictions imposed: (1) all assignments have an e-value lower than 10^−4^; and (2) no other assignment on the same region of the gene is made with a lower e-value. Table S1 shows the genomes and their coverage by assignments.

### PSI-BLAST assignments.

The second set consisted of assignments obtained by PSI-BLAST [5] searches on the same 150 genomes. Sequences with less than 95% sequence identity from the ASTRAL [32] database were used to search for structural domains in a nonredundant database created from all genes in the 150-genome set. PSI-BLAST was used with a SEG filter and an e-value cutoff of 1 × 10^−5^ for inclusion in the PSSM. Assignments with an e-value smaller than 1.0 were included after the final run. The e-values for the assignments were taken from the PSI-BLAST run in which the assignment first falls below 1 × 10^−5^ (i.e., when it is not yet included for scoring in the PSSM). Note that there was no check for overlap of assignments within a gene. In contrast with SUPERFAMILY, a repeat of a domain from the same superfamily within a gene was counted as a single copy.

### Phylogenies.

Phylogenies of the genomes were created using the SUPERFAMILY occurrence data (the number of copies were not included). A neighbour-joining algorithm was used to create a tree. The branch lengths were then normalised so that all leaves were at an equal distance from the root (1.0), following the method used by Winstanley and coworkers [17].

### Occurrences.

An occurrence is in this study defined as the occurrence of a superfamily on a genome. An occurrence can therefore be caused by more than one assignment. The e-value of an occurrence is defined as the lowest e-value in the set of assignments covering the occurrence.

### Isolated occurrences—gains at leaf level.

A parsimony algorithm is used to find isolated occurrences in a pattern given the phylogeny. This minimises the number of loss and gain events for a superfamily in the species tree [17,33,34]; a detailed description of the algorithm can be found in [33]. Isolated occurrences are identified as occurrences that create a gain at leaf level. The algorithm used a gain penalty that was twice the size of the loss penalty in order to take a high number of false negatives into account. Experimentation with a lower relative gain penalty showed only a small increase in isolated occurrences. This indicates the technique is relatively robust against false negatives. The parsimony algorithm was implemented in Java (J2SE 5.0; http://java.sun.com).

### Potential gains at leaf level.

A potential gain at leaf level is defined as a genome without an occurrence for a given superfamily that would cause a gain at leaf level if an occurrence were added to the existing pattern. To calculate potential gains at leaf level, the parsimony algorithm is run for every genome without an occurrence in the pattern.

### Identifying isolated occurrences using a base pattern.

A base pattern is created from assignments with an e-value below a given threshold. Subsequently, the parsimony algorithm is run on the base pattern, and potential gains at leaf level are predicted. A set of isolated occurrences can be found as the union of the set of (1) occurrences within the base pattern which cause a gain at leaf level; and (2) all occurrences caused by assignments above the e-value threshold, with a potential gain at leaf level.

### Distance to source.

The distance to source for an occurrence is the age of the youngest common ancestor between the occurrence and any genome containing the source domain. A genome is said to contain a source sequence for a SCOP superfamily if it covers 80% of its length and has at least 95% sequence identity. Simple BLAST searches were used to identify the source sequences. No source sequences could be identified for a few superfamilies on our set of genomes.

### Cluster distance.

The cluster distance reflects how “far away” an occurrence is from any other occurrence within a pattern, given the phylogeny. The cluster distance is calculated as the sum of distances to every occurrence in the tree divided by the distance to every leaf in the tree. This score was subsequently divided by the average score for each occurrence in the pattern so that the average cluster distance of a pattern became 1.0. A cluster score of 1.0 indicates average clustering; a score of <1.0 indicates tighter clustering. Mediation by the average distance to each leaf was used, since some leaves in the tree lie in tighter clusters and would generally produce lower scores without mediation.

### Consensus.

A consensus occurrence is an occurrence that is identified by both SUPERFAMILY and PSI-BLAST. Note that the occurrence does not necessarily have to be caused by assignments to the same gene.

### Significance test for structural classes.

A simple sampling procedure was used to determine if the proportion of false positives for a structural class deviates significantly from the overall false positive rate in Table 2. The ratio of predicted false positives was calculated for 500 random samples of the occurrence patterns. For each sample, the number of random genome entries was chosen to match the class size. The resulting distribution of sampled false positives rates was used to determine if the rate for each structural class was significantly lower, falling within the lowest 1% of the sampled distribution, or significantly higher, falling within highest 1%. The number of genome entries for a class depends on the number of superfamilies and ranges from almost 6,000 entries (multidomain) to just over 50,000 entries (alpha/beta).

### PSI-BLAST overlapping regions.

Overlapping regions of assignments were identified as assignments with a stronger assignment to a different superfamily on the same gene region and with at least 50% of the weaker assignment covered by the stronger assignment. Overlapping occurrences are occurrences that would be taken out of the dataset if only the strongest assignment within a region was retained.

### Figures.

All figures are plotted using R [35] except Figure 1, which was created by a modified version of a Java applet (http://www.stats.ox.ac.uk/∼abeln/howold). Linear regression was also performed using R. Figure 2 is plotted using a kernel estimate for the density function; the amplitude is then multiplied by the number of data elements in the set to obtain an approximate frequency.

## Supporting Information

Figure S1 Correlation between e-Values and the Distance to a Source Sequence Correlation between e-values and the distance to a source sequence for both SUPERFAMILY (A) and PSI-BLAST (B). The distance to a source sequence for an occurrence is calculated as the distance to the youngest common ancestor between the occurrence and a genome containing a source sequence. The correlation shown is very weak (r-squared is 0.05 for SUPERFAMILY and 0.15 for PSI-BLAST).
(4.0 MB PDF)

Figure S2 The Frequency of e-Values for Different Cluster Distances (A,B) Show that occurrences with a relatively high cluster distance (>1.01) have a distribution similar to the assignment distribution for most of the e-value spectrum, since the ranked distribution is near uniform. Only the right-hand side of the distribution shows a drastic increase in frequency compared with the reference distribution. This sudden increase is once again likely to be caused by false positives. Moreover, occurrences with a cluster distance >1.20 give a very similar distribution of e-values to occurrences with a gain at leaf level.
(9 KB PDF)

Table S1 Structural Assignments to the 150 Genomes This table shows all 150 genomes for which structural assignments are made. The keys to the genomes as used in Figure 1 are given. The coverage of structural assignments is shown for each genome by the number of genes for which at least one assignment could be made. The last column shows the number of additional copies in the SUPERFAMILY data with respect to PSI-BLAST. For the PSI-BLAST data, any superfamily domain that is repeated within the same gene is counted as a single copy, for the SUPERFAMILY data each repeated superfamily is counted separately.
(294 KB DOC)

## Abbreviations

HGT: horizontal gene transfer
HMM: hidden Markov model
PSSM: position-specific scoring matrix
