# Supplementary material for: Using Phylogeny to Improve Genome-Wide Distant Homology Recognition
Source: PLoS Comput Biol. 2007 Jan 19;3(1):e3. doi: 10.1371/journal.pcbi.0030003 (PMC1779300; doi:10.1371/journal.pcbi.0030003)
Supplement: Supplementary file 1 [file pcbi.0030003.sg001.pdf]

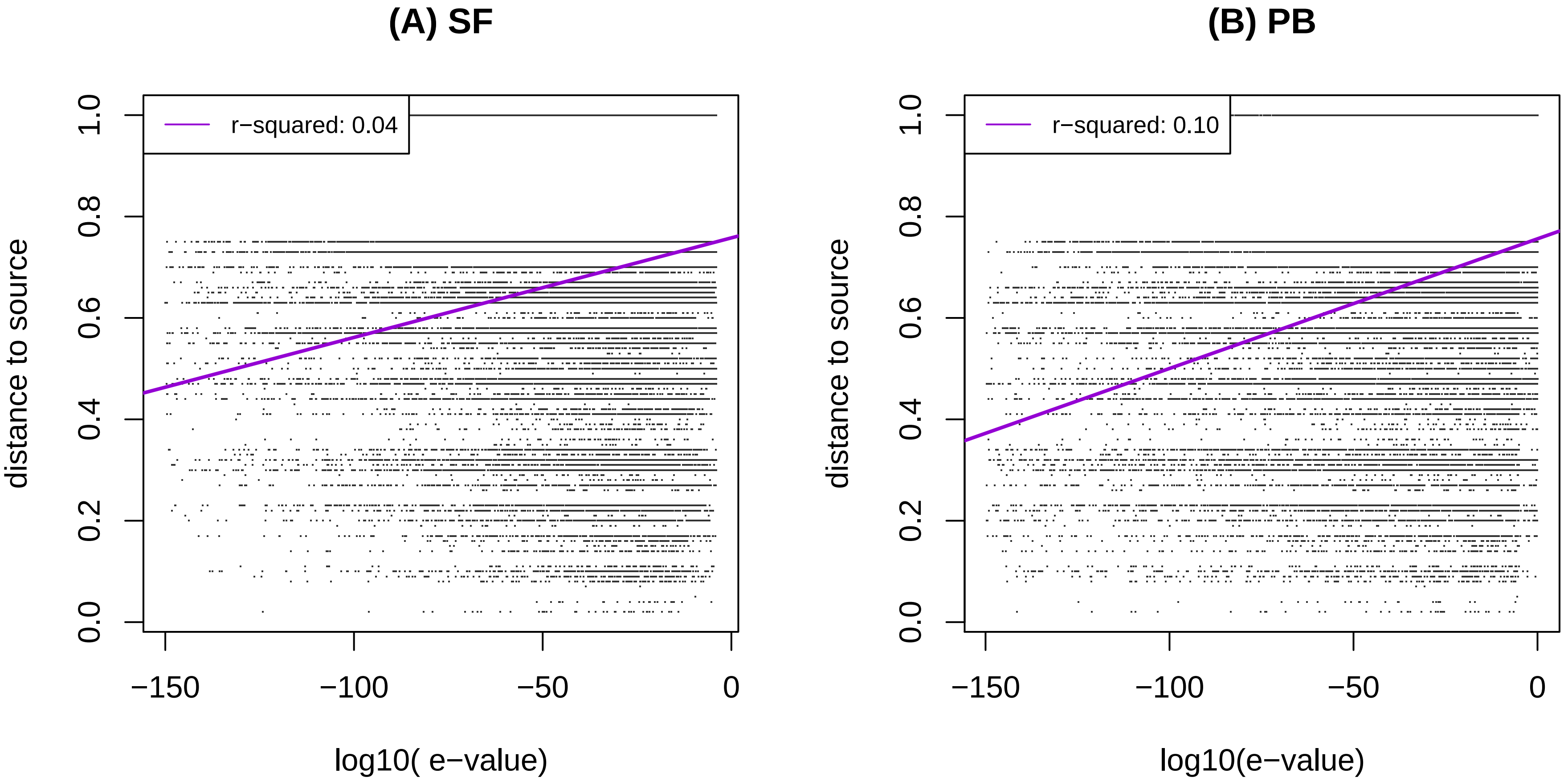

(A) SF
r−squared: 0.04
distance to source
log10(e−value)
0.0
0.2
0.4
0.6
0.8
1.0
−150
−100
−50
0
(B) PB
r−squared: 0.10
distance to source
log10(e−value)
