# Supplementary material for: Using Phylogeny to Improve Genome-Wide Distant Homology Recognition
Source: PLoS Comput Biol. 2007 Jan 19;3(1):e3. doi: 10.1371/journal.pcbi.0030003 (PMC1779300; doi:10.1371/journal.pcbi.0030003)
Supplement: Supplementary file 2 [file pcbi.0030003.sg002.pdf]

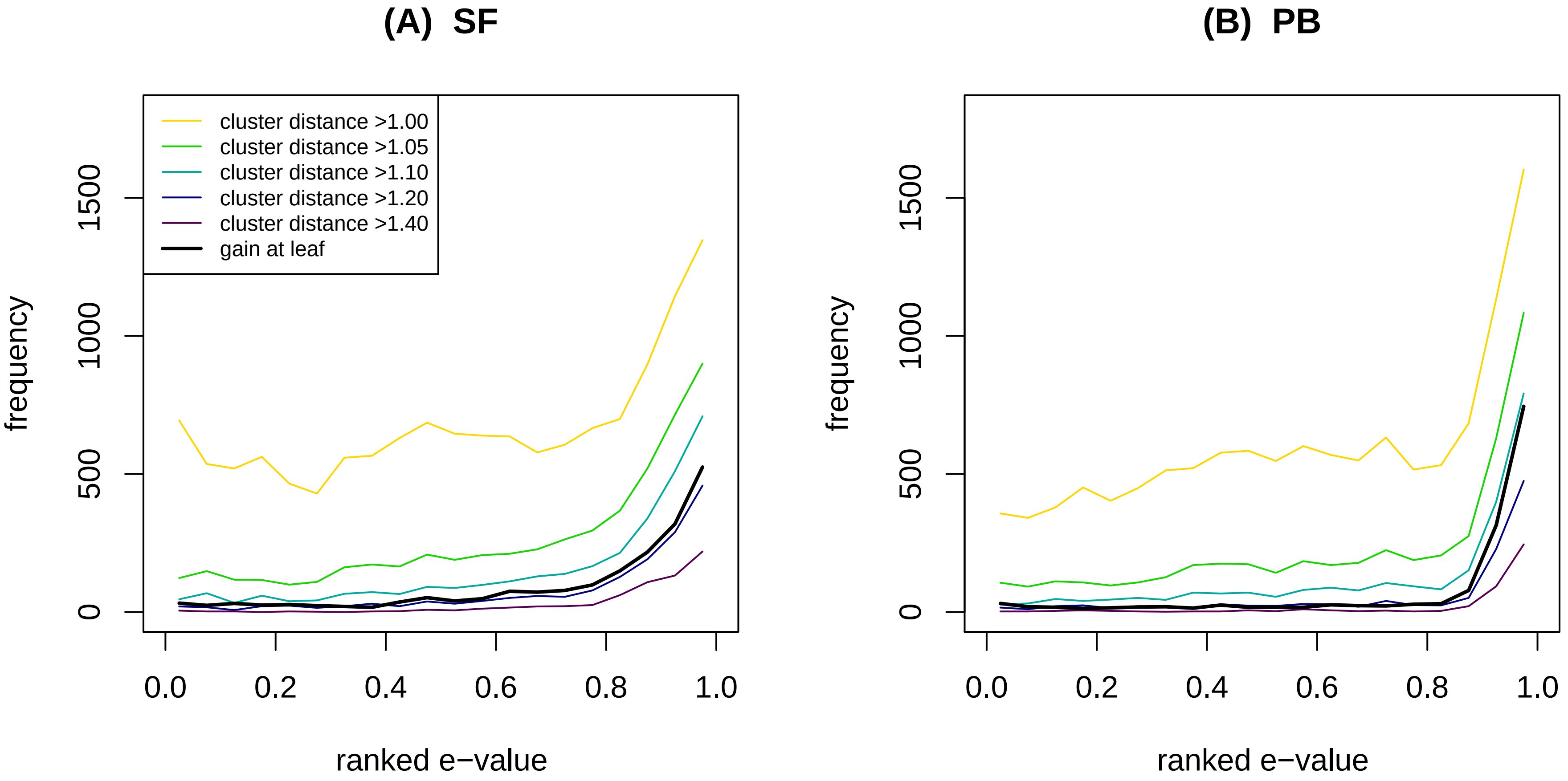

(A) SF
(B) PB
cluster distance >1.00
cluster distance >1.05
cluster distance >1.10
cluster distance >1.20
cluster distance >1.40
gain at leaf
frequency
0
500
1000
1500
0.0
0.2
0.4
0.6
0.8
1.0
ranked e−value
