# Supplementary material for: Using Phylogeny to Improve Genome-Wide Distant Homology Recognition
Source: PLoS Comput Biol. 2007 Jan 19;3(1):e3. doi: 10.1371/journal.pcbi.0030003 (PMC1779300; doi:10.1371/journal.pcbi.0030003)
Supplement: Supplementary file 3 [file pcbi.0030003.st001.doc]

| keys | names | genes | assignments SF | genes covered SF | % | assignments PB | genes covered PB | % | repeated domains SF |
| --- | --- | --- | --- | --- | --- | --- | --- | --- | --- |
| ACrTh_Aper | Aeropyrum_pernix | 1841 | 1257 | 945 | 51 | 827 | 634 | 34 | 88 |
| ACrTh_Paer | Pyrobaculum_aerophilum | 2605 | 1659 | 1259 | 48 | 1087 | 824 | 32 | 111 |
| ACrTh_Ssol | Sulfolobus_solfataricus | 2977 | 2098 | 1588 | 53 | 1334 | 1024 | 34 | 113 |
| ACrTh_Stok | Sulfolobus_tokodaii | 2825 | 1965 | 1486 | 53 | 1266 | 969 | 34 | 107 |
| AEuAr_Aful | Archaeoglobus_fulgidus | 2420 | 2014 | 1468 | 61 | 1369 | 1031 | 43 | 149 |
| AEuHa_Hsp. | Halobacterium | 2075 | 1657 | 1181 | 57 | 1089 | 806 | 39 | 107 |
| AEuMe_Mace | Methanosarcina_acetivorans | 4540 | 3575 | 2479 | 55 | 2220 | 1645 | 36 | 455 |
| AEuMe_Mjan | Methanocaldococcus_jannaschii | 1786 | 1399 | 1027 | 58 | 1025 | 757 | 42 | 98 |
| AEuMe_Mkan | Methanopyrus_kandleri | 1687 | 1251 | 916 | 54 | 942 | 701 | 42 | 89 |
| AEuMe_Mmar | Methanococcus_maripaludis | 1722 | 1380 | 1017 | 59 | 1023 | 778 | 45 | 80 |
| AEuMe_Mmaz | Methanosarcina_mazei_Go | 3371 | 2614 | 1887 | 56 | 1787 | 1328 | 39 | 254 |
| AEuMe_Mthe | Methanothermobacter_thermautotrophicus | 1873 | 1573 | 1128 | 60 | 1128 | 835 | 45 | 120 |
| AEuTh_Paby | Pyrococcus_abyssi | 1896 | 1533 | 1131 | 60 | 1119 | 812 | 43 | 84 |
| AEuTh_Pfur | Pyrococcus_furiosus | 2125 | 1670 | 1229 | 58 | 1216 | 875 | 41 | 86 |
| AEuTh_Phor | Pyrococcus_horikoshii | 1955 | 1473 | 1078 | 55 | 1067 | 767 | 39 | 84 |
| AEuTh_Ptor | Picrophilus_torridus | 1535 | 1250 | 930 | 61 | 890 | 675 | 44 | 68 |
| AEuTh_Taci | Thermoplasma_acidophilum | 1482 | 1286 | 938 | 63 | 893 | 660 | 45 | 72 |
| AEuTh_Tvol | Thermoplasma_volcanium | 1499 | 1270 | 934 | 62 | 898 | 678 | 45 | 68 |
| BAcAc_Blon | Bifidobacterium_longum | 1727 | 1571 | 1092 | 63 | 1119 | 799 | 46 | 94 |
| BAcAc_Cdip | Corynebacterium_diphtheriae | 1259 | 1891 | 1303 | 103 | 679 | 508 | 40 | 142 |
| BAcAc_Ceff | Corynebacterium_efficiens | 2950 | 2371 | 1677 | 57 | 1561 | 1149 | 39 | 168 |
| BAcAc_Cglu | Corynebacterium_glutamicum | 3057 | 2456 | 1739 | 57 | 1601 | 1205 | 39 | 172 |
| BAcAc_Lxyl | Leifsonia_xyli | 2030 | 1741 | 1228 | 60 | 1128 | 831 | 41 | 105 |
| BAcAc_Mavi | Mycobacterium_avium | 4350 | 3990 | 2846 | 65 | 2403 | 1849 | 43 | 307 |
| BAcAc_Mbov | Mycobacterium_bovis | 3920 | 3336 | 2326 | 59 | 2053 | 1514 | 39 | 240 |
| BAcAc_Mlep | Mycobacterium_leprae | 1605 | 1523 | 1002 | 62 | 1059 | 738 | 46 | 118 |
| BAcAc_Mtub | Mycobacterium_tuberculosis_H37Rv | 3991 | 3380 | 2350 | 59 | 2068 | 1528 | 38 | 243 |
| BAcAc_Pacn | Propionibacterium_acnes | 2297 | 1988 | 1392 | 61 | 1411 | 1007 | 44 | 126 |
| BAcAc_Save | Streptomyces_avermitilis | 7577 | 6683 | 4598 | 61 | 3901 | 2922 | 39 | 538 |
| BAcAc_Scoe | Streptomyces_coelicolor | 7769 | 6795 | 4753 | 61 | 3980 | 2999 | 39 | 447 |
| BAqAq_Aaeo | Aquifex_aeolicus | 1529 | 1515 | 1044 | 68 | 1166 | 828 | 54 | 99 |
| BBaBa_Bthe | Bacteroides_thetaiotaomicron | 4778 | 4036 | 2729 | 57 | 2572 | 1872 | 39 | 233 |
| BBaBa_Pgin | Porphyromonas_gingivalis | 1909 | 1556 | 1063 | 56 | 1055 | 749 | 39 | 101 |
| BChCh_Ctep | Chlorobium_tepidum | 2252 | 1840 | 1283 | 57 | 1380 | 989 | 44 | 116 |
| BCyCh_Gvio | Gloeobacter_violaceus | 4430 | 3634 | 2544 | 57 | 2431 | 1727 | 39 | 264 |
| BCyCh_Ssp* | Synechocystis | 3167 | 2785 | 1903 | 60 | 1866 | 1335 | 42 | 215 |
| BCyCh_Ssp. | Synechococcus | 2517 | 1962 | 1405 | 56 | 1288 | 983 | 39 | 131 |
| BCyCh_Telo | Thermosynechococcus_elongatus | 2475 | 2172 | 1477 | 60 | 1450 | 1056 | 43 | 167 |
| BCyNo_Nsp. | Nostoc | 5366 | 4540 | 3007 | 56 | 2925 | 2028 | 38 | 412 |
| BCyPr_Pmar | Prochlorococcus_marinus | 1882 | 1440 | 1023 | 54 | 1042 | 757 | 40 | 91 |
| BDeDe_Drad | Deinococcus_radiodurans | 2997 | 2438 | 1770 | 59 | 1641 | 1239 | 41 | 153 |
| BDeDe_Tthe | Thermus_thermophilus | 1982 | 1868 | 1294 | 65 | 1375 | 998 | 50 | 136 |
| BFiBa_Bant | Bacillus_anthracis | 5311 | 3715 | 2702 | 51 | 2579 | 1943 | 37 | 202 |
| BFiBa_Bcer | Bacillus_cereus | 5234 | 3848 | 2797 | 53 | 2653 | 2003 | 38 | 215 |
| BFiBa_Bhal | Bacillus_halodurans | 4066 | 3381 | 2379 | 59 | 2330 | 1722 | 42 | 221 |
| BFiBa_Bsub | Bacillus_subtilis | 4105 | 3401 | 2339 | 57 | 2408 | 1764 | 43 | 275 |
| BFiBa_Bthu | Bacillus_thuringiensis_serovar_konkukian | 5117 | 3873 | 2797 | 55 | 2674 | 2014 | 39 | 220 |
| BFiBa_Linn | Listeria_innocua_Clip | 2968 | 2552 | 1795 | 60 | 1763 | 1298 | 44 | 177 |
| BFiBa_Lmon | Listeria_monocytogenes_EGD-e | 2846 | 2578 | 1809 | 64 | 1830 | 1333 | 47 | 179 |
| BFiBa_Oihe | Oceanobacillus_iheyensis | 3500 | 2919 | 2088 | 60 | 2079 | 1540 | 44 | 172 |
| BFiBa_Saur | Staphylococcus_aureus | 2697 | 2239 | 1568 | 58 | 1590 | 1154 | 43 | 142 |
| BFiBa_Sepi | Staphylococcus_epidermidis | 2419 | 1972 | 1378 | 57 | 1432 | 1025 | 42 | 125 |
| BFiCl_Cace | Clostridium_acetobutylicum | 3672 | 2990 | 2139 | 58 | 2046 | 1521 | 41 | 203 |
| BFiCl_Cper | Clostridium_perfringens | 2660 | 2310 | 1609 | 60 | 1700 | 1209 | 45 | 155 |
| BFiCl_Ctet | Clostridium_tetani | 2373 | 2065 | 1418 | 60 | 1468 | 1028 | 43 | 117 |
| BFiCl_Tten | Thermoanaerobacter_tengcongensis | 2588 | 2280 | 1571 | 61 | 1674 | 1161 | 45 | 152 |
| BFiLa_Efae | Enterococcus_faecalis | 3113 | 2415 | 1688 | 54 | 1677 | 1237 | 40 | 153 |
| BFiLa_Ljoh | Lactobacillus_johnsonii | 1821 | 1563 | 1109 | 61 | 1075 | 782 | 43 | 85 |
| BFiLa_Llac | Lactococcus_lactis | 2321 | 1959 | 1400 | 60 | 1316 | 980 | 42 | 113 |
| BFiLa_Lpla | Lactobacillus_plantarum | 3009 | 2503 | 1805 | 60 | 1681 | 1272 | 42 | 125 |
| BFiLa_Saga | Streptococcus_agalactiae | 2124 | 1808 | 1279 | 60 | 1246 | 913 | 43 | 104 |
| BFiLa_Smut | Streptococcus_mutans | 1960 | 1777 | 1252 | 64 | 1224 | 894 | 46 | 111 |
| BFiLa_Spne | Streptococcus_pneumoniae | 2094 | 1716 | 1224 | 58 | 1225 | 903 | 43 | 106 |
| BFiLa_Spy* | Streptococcus_pyogenes_M1GAS | 1697 | 1525 | 1064 | 63 | 1066 | 773 | 46 | 95 |
| BFiLa_Spyo | Streptococcus_pyogenes_MGAS10394 | 1886 | 1593 | 1117 | 59 | 1095 | 798 | 42 | 97 |
| BFuFu_Fnuc | Fusobacterium_nucleatum | 2067 | 1666 | 1200 | 58 | 1128 | 844 | 41 | 106 |
| BPrAl_Bjap | Bradyrhizobium_japonicum | 8317 | 7068 | 5031 | 60 | 4249 | 3295 | 40 | 459 |
| BPrAl_Bmel | Brucella_melitensis | 3198 | 2851 | 2017 | 63 | 1882 | 1423 | 44 | 172 |
| BPrAl_Bsui | Brucella_suis | 3271 | 2684 | 1883 | 58 | 1826 | 1375 | 42 | 162 |
| BPrAl_Ccre | Caulobacter_crescentus | 3737 | 3318 | 2333 | 62 | 2074 | 1550 | 41 | 198 |
| BPrAl_Mlot | Mesorhizobium_loti | 6743 | 5700 | 4094 | 61 | 3526 | 2729 | 40 | 321 |
| BPrAl_Rpal | Rhodopseudomonas_palustris | 4813 | 4403 | 3082 | 64 | 2729 | 2080 | 43 | 321 |
| BPrAl_Rsph | Rhodobacter_sphaeroides | 11584 | 3411 | 2410 | 21 | 2150 | 1617 | 14 | 210 |
| BPrAl_Smel | Sinorhizobium_meliloti | 3341 | 3065 | 2159 | 65 | 2011 | 1497 | 45 | 184 |
| BPrBe_Bbro | Bordetella_bronchiseptica | 4994 | 4763 | 3395 | 68 | 2896 | 2225 | 45 | 285 |
| BPrBe_Bpar | Bordetella_parapertussis | 4185 | 4114 | 2936 | 70 | 2518 | 1920 | 46 | 244 |
| BPrBe_Bper | Bordetella_pertussis_Tohama | 3436 | 3602 | 2433 | 71 | 2032 | 1516 | 44 | 199 |
| BPrBe_Cvio | Chromobacterium_violaceum | 4407 | 3839 | 2594 | 59 | 2423 | 1793 | 41 | 286 |
| BPrBe_Neur | Nitrosomonas_europaea | 2461 | 2284 | 1570 | 64 | 1486 | 1071 | 44 | 164 |
| BPrBe_Nmen | Neisseria_meningitidis | 2079 | 1674 | 1154 | 56 | 1225 | 900 | 43 | 111 |
| BPrBe_Rsol | Ralstonia_solanacearum | 3440 | 3023 | 2135 | 62 | 2015 | 1502 | 44 | 176 |
| BPrDe_Bbac | Bdellovibrio_bacteriovorus | 3587 | 2633 | 1873 | 52 | 1673 | 1257 | 35 | 181 |
| BPrDe_Dpsy | Desulfotalea_psychrophila_LSv | 3116 | 2482 | 1695 | 54 | 1698 | 1242 | 40 | 173 |
| BPrDe_Dvul | Desulfovibrio_vulgaris | 3379 | 2674 | 1762 | 52 | 1765 | 1282 | 38 | 221 |
| BPrDe_Gsul | Geobacter_sulfurreducens | 3446 | 3227 | 2134 | 62 | 2147 | 1544 | 45 | 240 |
| BPrEp_Cjej | Campylobacter_jejuni | 1629 | 1395 | 995 | 61 | 1010 | 766 | 47 | 85 |
| BPrEp_Hhep | Helicobacter_hepaticus | 1875 | 1420 | 1012 | 54 | 965 | 733 | 39 | 88 |
| BPrEp_Hpyl | Helicobacter_pylori | 1576 | 1177 | 828 | 53 | 840 | 632 | 40 | 79 |
| BPrEp_Wsuc | Wolinella_succinogenes | 2043 | 1881 | 1293 | 63 | 1238 | 931 | 46 | 130 |
| BPrGa_Asp. | Acinetobacter | 3325 | 2857 | 1991 | 60 | 1971 | 1451 | 44 | 188 |
| BPrGa_Cbur | Coxiella_burnetii | 2010 | 1509 | 1021 | 51 | 1058 | 731 | 36 | 98 |
| BPrGa_Ecar | Erwinia_carotovora | 4472 | 3887 | 2682 | 60 | 2636 | 1946 | 44 | 292 |
| BPrGa_Ecol | Escherichia_coli | 4237 | 3906 | 2668 | 63 | 2698 | 1969 | 46 | 272 |
| BPrGa_Hduc | Haemophilus_ducreyi | 1717 | 1370 | 957 | 56 | 1037 | 739 | 43 | 90 |
| BPrGa_Hinf | Haemophilus_influenzae_Rd | 1657 | 1633 | 1135 | 68 | 1208 | 872 | 53 | 107 |
| BPrGa_Paer | Pseudomonas_aeruginosa | 5567 | 5364 | 3582 | 64 | 3346 | 2482 | 45 | 412 |
| BPrGa_Plum | Photorhabdus_luminescens | 4683 | 3799 | 2585 | 55 | 2354 | 1732 | 37 | 312 |
| BPrGa_Pmul | Pasteurella_multocida | 2015 | 1937 | 1361 | 68 | 1374 | 1012 | 50 | 124 |
| BPrGa_Pput | Pseudomonas_putida | 5350 | 4900 | 3290 | 61 | 3046 | 2251 | 42 | 342 |
| BPrGa_Psyr | Pseudomonas_syringae_pv_tomato | 3971 | 4795 | 3238 | 82 | 2111 | 1593 | 40 | 380 |
| BPrGa_Sent | Salmonella_enterica | 4395 | 3649 | 2548 | 58 | 2517 | 1862 | 42 | 219 |
| BPrGa_Sfle | Shigella_flexneri_2a | 4182 | 3503 | 2496 | 60 | 2364 | 1731 | 41 | 203 |
| BPrGa_Sone | Shewanella_oneidensis | 4324 | 3635 | 2486 | 57 | 2282 | 1668 | 39 | 266 |
| BPrGa_Styp | Salmonella_typhimurium | 4425 | 3875 | 2682 | 61 | 2685 | 1980 | 45 | 238 |
| BPrGa_Vcho | Vibrio_cholerae_O1_biovar_eltor | 3835 | 3224 | 2189 | 57 | 2095 | 1530 | 40 | 213 |
| BPrGa_Vpar | Vibrio_parahaemolyticus | 4756 | 3985 | 2745 | 58 | 2504 | 1859 | 39 | 259 |
| BPrGa_Vvu* | Vibrio_vulnificus_CMCP6 | 4488 | 3958 | 2708 | 60 | 2515 | 1840 | 41 | 250 |
| BPrGa_Vvul | Vibrio_vulnificus_YJ016 | 4955 | 4051 | 2777 | 56 | 2580 | 1888 | 38 | 260 |
| BPrGa_Xaxo | Xanthomonas_axonopodis_pv_citri | 4312 | 3813 | 2654 | 62 | 2460 | 1799 | 42 | 236 |
| BPrGa_Xcam | Xanthomonas_campestris_pv_campestris | 4181 | 3701 | 2573 | 62 | 2373 | 1736 | 42 | 232 |
| BPrGa_Xfas | Xylella_fastidiosa_9a5c | 2766 | 1893 | 1296 | 47 | 1335 | 959 | 35 | 136 |
| BPrGa_Ypes | Yersinia_pestis | 3885 | 3417 | 2321 | 60 | 2282 | 1656 | 43 | 245 |
| BPrGa_Ypse | Yersinia_pseudotuberculosis | 3901 | 3456 | 2353 | 60 | 2353 | 1704 | 44 | 250 |
| BSpSp_Lint | Leptospira_interrogans_serovar_Lai | 4727 | 2665 | 1931 | 41 | 1568 | 1200 | 25 | 186 |
| BThTh_Tmar | Thermotoga_maritima | 1858 | 1701 | 1193 | 64 | 1272 | 887 | 48 | 99 |
| EAlAp_Pfal | Plasmodium_falciparum | 5417 | 3008 | 2273 | 42 | 1792 | 1409 | 26 | 269 |
| EAlAp_Pyoe | Plasmodium_yoelii | 7861 | 2976 | 2238 | 28 | 1619 | 1259 | 16 | 261 |
| EFuAs_Agos | Ashbya_gossypii | 4718 | 3689 | 2611 | 55 | 2275 | 1751 | 37 | 373 |
| EFuAs_Anid | Aspergillus_nidulans | 9541 | 7733 | 5291 | 55 | 4497 | 3303 | 35 | 725 |
| EFuAs_Calb | Candida_albicans | 12017 | 4339 | 3185 | 27 | 5039 | 3906 | 33 | 391 |
| EFuAs_Cgla | Candida_glabrata | 5181 | 3949 | 2804 | 54 | 2438 | 1875 | 36 | 409 |
| EFuAs_Dhan | Debaromyces_hansenii | 6317 | 4563 | 3350 | 53 | 2705 | 2108 | 33 | 418 |
| EFuAs_Fgra | Fusarium_graminearum | 11640 | 8379 | 6061 | 52 | 4876 | 3758 | 32 | 730 |
| EFuAs_Klac | Kluyveromyces_lactis | 5327 | 3897 | 2781 | 52 | 2435 | 1860 | 35 | 393 |
| EFuAs_Kwal | Kluyveromyces_waltii | 5214 | 3822 | 2754 | 53 | 2321 | 1791 | 34 | 390 |
| EFuAs_Mgri | Magnaporthe_grisea | 11109 | 7270 | 5184 | 47 | 3990 | 3119 | 28 | 611 |
| EFuAs_Ncra | Neurospora_crassa | 10620 | 5959 | 4203 | 40 | 3497 | 2692 | 25 | 573 |
| EFuAs_Sbay | Saccharomyces_bayanus | 18770 | 4490 | 3319 | 18 | 2586 | 2029 | 11 | 402 |
| EFuAs_Scer | Saccharomyces_cerevisiae | 11686 | 4704 | 3303 | 28 | 2698 | 2081 | 18 | 465 |
| EFuAs_Smik | Saccharomyces_mikatae | 18032 | 4491 | 3320 | 18 | 2600 | 2041 | 11 | 393 |
| EFuAs_Spar | Saccharomyces_paradoxus | 17878 | 4509 | 3242 | 18 | 2695 | 2094 | 12 | 436 |
| EFuAs_Spom | Schizosaccharomyces_pombe | 5000 | 4262 | 2949 | 59 | 2487 | 1923 | 38 | 455 |
| EFuAs_Ylip | Yarrowia_lipolytica | 6521 | 4709 | 3413 | 52 | 2825 | 2186 | 34 | 437 |
| EFuBa_Umay | Ustilago_maydis | 6522 | 4771 | 3348 | 51 | 2911 | 2217 | 34 | 452 |
| EMeAr_Agam | Anopheles_gambiae | 15212 | 13516 | 8211 | 54 | 6551 | 4992 | 33 | 3106 |
| EMeAr_Dmel | Drosophila_melanogaster | 10538 | 12607 | 7376 | 70 | 5560 | 3949 | 37 | 3080 |
| EMeCh_Cint | Ciona_intestinalis | 21574 | 16247 | 9119 | 42 | 13057 | 8611 | 40 | 4566 |
| EMeCh_Drer | Danio_rerio | 29663 | 26162 | 14998 | 51 | 17261 | 13029 | 44 | 6541 |
| EMeCh_Frub | Fugu_rubripes | 33003 | 25061 | 13125 | 40 | 22148 | 14995 | 45 | 7162 |
| EMeCh_Ggal | Gallus_gallus | 28416 | 19093 | 10289 | 36 | 16538 | 11600 | 41 | 5260 |
| EMeCh_Hsap | Homo_sapiens | 33869 | 29519 | 14252 | 42 | 19590 | 14266 | 42 | 10326 |
| EMeCh_Mmus | Mus_musculus | 31535 | 30578 | 16229 | 51 | 16753 | 12467 | 40 | 9568 |
| EMeCh_Ptro | Pan_troglodytes | 39648 | 23599 | 12565 | 32 | 16280 | 12735 | 32 | 7458 |
| EMeCh_Rnor | Rattus_norvegicus | 32543 | 27524 | 14781 | 45 | 19111 | 13977 | 43 | 8263 |
| EMeCh_Xtro | Xenopus_tropicalis | 52786 | 35822 | 19398 | 37 | 33334 | 23046 | 44 | 9853 |
| EMeNe_Cbri | Caenorhabditis_briggsae | 19334 | 15143 | 10018 | 52 | 5710 | 4529 | 23 | 2717 |
| EMeNe_Cele | Caenorhabditis_elegans | 21124 | 15855 | 10608 | 50 | 6699 | 5294 | 25 | 2678 |
| EMyDi_Ddis | Dictyostelium_discoideum | 13049 | 8804 | 6167 | 47 | 4456 | 3459 | 27 | 930 |
| EViSt_Atha | Arabidopsis_thaliana | 28860 | 22072 | 15636 | 54 | 12412 | 10224 | 35 | 2215 |
| EViSt_Osat | Oryza_sativa | 61250 | 29061 | 20588 | 34 | 17742 | 14407 | 24 | 2595 |
| all | all genomes | 1032104 | 758437 | 490265 | 48 | 497183 | 368459 | 36 | 116200 |
